# Experimental infection of non-immunosuppressed and immunosuppressed goats reveals differential pathogenesis of *Babesia aktasi* n. sp.

**DOI:** 10.3389/fcimb.2023.1277956

**Published:** 2023-11-02

**Authors:** Sezayi Ozubek, Mehmet Can Ulucesme, Reginaldo G. Bastos, Heba F. Alzan, Jacob M. Laughery, Carlos E. Suarez, Munir Aktas

**Affiliations:** ^1^ Department of Parasitology, Faculty of Veterinary Medicine, University of Firat, Elazig, Türkiye; ^2^ Animal Disease Research Unit, United States Department of Agricultural (USDA), Agricultural Research Service, Pullman, WA, United States; ^3^ Department of Veterinary Microbiology and Pathology, College of Veterinary Medicine, Washington State University, Pullman, WA, United States; ^4^ Parasitology and Animal Diseases Department, National Research Center, Giza, Egypt

**Keywords:** *Babesia aktasi* n. sp., experimental infection, goat, immunosuppression, pathogenicity

## Abstract

Babesiosis is an acute and persistent tick-borne disease caused by protozoan parasites of the genus *Babesia*. These hemoparasites affect vertebrates globally, resulting in symptoms such as high fever, anemia, jaundice, and even death. Advancements in molecular parasitology revealed new *Babesia* species/genotypes affecting sheep and goats, including *Babesia aktasi* n. sp., which is highly prevalent in goats from Turkiye’s Mediterranean region. The objective of this study was to investigate the pathogenesis of *B. aktasi* infection in immunosuppressed (n=7) and non-immunosuppressed (n=6) goats. These animals were experimentally infected with fresh *B. aktasi* infected blood, and their clinical signs, hematological and serum biochemical parameters were monitored throughout the infection. The presence of parasites in the blood of immunosuppressed goats was detected by microscopic examination between 4 and 6 days after infection, accompanied by fever and increasing parasitemia. Goats that succumbed acute disease exhibited severe clinical signs, such as anemia, hemoglobinuria, and loss of appetite. However, the goats that survived showed milder clinical signs. In the non-immunosuppressed group, piroplasm forms of *B. aktasi* were observed in the blood within 2-5 days after inoculation, but with low (0.01-0.2%) parasitemia. Although these goats showed loss of appetite, typical signs of babesiosis were absent except for increased body temperature. Hematological analysis revealed significant decreases in the levels of red blood cells, leukocytes and platelet values post-infection in immunosuppressed goats, while no significant hematological changes were observed in non-immunosuppressed goats. In addition, serum biochemical analysis showed elevated transaminase liver enzymes levels, decreased glucose, and lower total protein values in the immunosuppressed group post-infection. *Babesia aktasi*, caused mild disease with minor clinical symptoms in non-immunosuppressed goats. However, in immunosuppressed goats, it exhibited remarkable pathogenicity, leading to severe clinical infections and death. In conclusion, this study provides valuable insights into the pathogenicity of the parasite and will serve as a foundation for future research aimed at developing effective prevention and control strategies against babesiosis in small ruminants. Further research is required to investigate the pathogenicity of *B. aktasi* in various goat breeds, other potential hosts, the vector ticks involved, and its presence in natural reservoirs.

## Introduction

Babesiosis, caused by protozoa of the genus *Babesia*, is one of the most common and economically important tick-borne diseases of domestic and wild ruminants worldwide (Homer et al., 2000). Babesiosis is an acute and chronic disease that can vary in severity depending on factors such as age, species, immunological status, presence of other pathogens, and genetic factors of the host. Typically, *Babesia* parasites invade and destroy vertebrate host erythrocytes and cause acute babesiosis characterized by high fever, anemia, icterus, hemoglobinuria, tachycardia, jaundice, weakness, lethargy, loss of appetite, abdominal pain, and a high mortality (Friedhoff, 1988). In contrast, chronic infections typically do not exhibit clinical signs but may negatively affect livestock production (Friedhoff, 1988; Homer et al., 2000). The disease is primarily transmitted by ticks belonging to the *Rhipicephalus*, *Dermacentor*, *Ixodes*, *Hyalomma* and *Haemaphysalis* genera (Guglielmone et al., 2010; Dantas-Torres, 2018; Gray et al., 2019). Currently, more than 100 species of *Babesia* have been identified worldwide that can infect humans, domestic and wild mammals, and most recently birds, and this number is likely to increase due to new research in other vertebrate hosts (Martínez-García et al., 2021). Of interest are the species *Babesia ovis*, *B. motasi*, and *B. crassa* that can cause babesiosis in sheep and goats (Liu et al., 2007; Ozubek and Aktas, 2017a; Schnittger et al., 2022), with *B. ovis* identified as the primary etiological agent of clinical babesiosis in these animals (Yeruham et al., 1998; Yeruham et al., 2001). Significant advances have been made in the field of molecular parasitology in the past two decades, leading to an increased interest in better defining blood protozoa belonging to the piroplasmid lineage. This has resulted in the identification of new *Babesia* species or genotypes that affect sheep and goats, including *Babesia* sp. Xinjiang, *Babesia lengau*-like, and *B. motasi*-like (*B. motasi* Lintan, *B. motasi* Tianzhu, *B. motasi* Hebei, and *B. motasi* Ningxian) (Liu et al., 2007; Guan et al., 2009; Niu et al., 2009; Bosman et al., 2010). The subspecies of *Babesia motasi* can be distinguished into two subtypes (*B. motasi* Lintanensis and *B. motasi* Hebeinensis) based on their distinct morphological, serological, pathogenic, genetic, and virulent characteristics (Wang et al., 2019; Wang et al., 2020; Wang et al., 2023). Using the PCR-based reverse line blot (RLB) method, we have recently discovered a new *Babesia* species in goats in Turkiye’s Mediterranean region, which is clearly distinct from the ovine *Babesia* species described to date (Ozubek and Aktas, 2017b). After being isolated from a naturally infected goat and having its genetic and morphological traits characterized, this newly identified *Babesia* species was named *Babesia aktasi* n. sp. (Ozubek et al., 2023). Following that, a large-scale survey in the Mediterranean region revealed that *B. aktasi* n. sp. has a high prevalence (22.5%) in goats (Ulucesme et al., 2023a). Comparisons of pathogenesis and virulence of *Babesia* species require systematic animal experiments and often the use of spleen-intact (non-immunosuppressed) and splenectomized (immunosuppressed) animals (Hasherni-Fesharki and Uilenberg, 1981; Habela et al., 1990; Guan et al., 2009). It is well known that the spleen plays an important role in the clearance of *Babesia*-infected erythrocytes. Thus, consistently, splenectomy reduces the host’s capacity to control the parasite, thereby allowing detection and sometimes significant expansion of parasite populations that were previously undetectable and potentially clinically relevant (Bach et al., 2005; Buffet et al., 2011; Lewis et al., 2019). This is particularly important for *Babesia* species such as *Babesia* sp. Xinjiang (Guan et al., 2009), *Babesia* sp. BQ1-Lintan (Guan et al., 2010) and *B. crassa* (Hasherni-Fesharki and Uilenberg, 1981) which have low pathogenicity, where comparing non-immunosuppressed and immunosuppressed animals is crucial. In a study with *Theileria haneyi*, which is a novel species infecting horses also known for its low pathogenicity, spleen intact and splenectomised groups were formed in experimental infections (Sears et al., 2022).

In fact, some studies have shown that dexamethasone treatment, in addition to splenectomy, leads to better results by further suppressing the immune system (Guan et al., 2001; Guan et al., 2009). In the *in vivo* isolation study, *B. aktasi* n. sp. was obtained from an apparently non-immunosuppressed goat through the suppression of its immune system. Subsequently, the isolated parasite was inoculated into another goat that had also undergone splenectomy and dexamethasone injection. Following inoculation, the second goat experienced a significant increase in body temperature and reached a parasitemia level of 10% on day three post-infection. Due to the severe acute symptoms of babesiosis, the goat was humanely euthanized four days post-infection (Ozubek et al., 2023). This previous study did not systematically investigate the pathogenicity of *B. aktasi* n. sp., highlighting the necessity for further research to enhance our understanding of this newly identified *Babesia* species. Conducting pathogenicity studies is essential to gain a comprehensive understanding of how *B. aktasi* n. sp. affects non-immunosuppressed goats, and its specific impact on immunosuppressed individuals. These studies can contribute significantly to our knowledge of the disease and help develop control interventions against small ruminant babesiosis. Therefore, this study was conducted to assess clinical, hematological, and biochemical alterations of goats experimentally infected with *B. aktasi* n. sp.

## Materials and methods

### Ethics statement

This study was carried out according to the regulations of animal and welfare issued by the Turkish legislation for the protection of animals. All animal experiments were approved by the Firat University, Animal Experiment Ethic Committee, protocol number 2018/100.

### *Babesia aktasi* n. sp. stabilate

*Babesia aktasi* n. sp., previously isolated from a naturally infected goat in the Mediterranean region of Turkiye, was utilized in this study (Ozubek et al., 2023). Briefly, one goat that was determined to be infected with *B. aktasi* n. sp. by nested PCR-based RLB in our previous field survey was splenectomized to increase circulating parasitemia and allow direct observation of the parasites in peripheral blood smears. When parasitemia reached 1.9%, 20 ml of blood from this goat was injected into another immunosuppressed goat that was free of tick-borne pathogens. After the detection of parasitemia (5% PPE) in the infected goat, 60 ml of blood was collected and divided into 5 ml aliquots, which were cryopreserved in 10% dimethyl sulfoxide (DMSO) solution. This *B. aktasi* n. sp. stabilate was used for experimental infections in the current study (Ozubek et al., 2023).

### Selection of experimental goats and splenectomy

Fourteen 6–8-month-old native local breed male goats were used in this study. Prior to the experiments, animals were shown to be negative for *Babesia*, *Theileria* and *Anaplasma* species by microscopy of peripheral blood smears and nested PCR-based RLB (Sevinc et al., 2007; Ulucesme et al., 2023a). During the experiment, the goats were housed in a closed pen at the veterinary medicine animal unit and received feed and water *ad libitum*. To prevent possible tick infestations, flumethrin 1% pour-on (Flugon® 1%, Vetaş, Turkiye) was applied to the goats according to the manufacturer’s recommendation, and acaricide application was continued at 21-day intervals throughout the experiment. The goats were divided into two groups: in group I, animals (n = 7) were immunosuppressed through a combination of splenectomy and dexamethasone application (Vetakort® 4 mg, Vetas, Turkiye; 20 mg/day for 3 days), while in group II, animals (n = 6) were spleen-intact and not immunosuppressed prior to *B. aktasi* n. sp. infection (Guan et al., 2009). Animals in group I underwent splenectomy performed at the Firat University Veterinary Hospital and were placed in separate compartments to ensure recovery for 2 weeks before proceeding with the experiment. The splenectomy was performed using standard surgical, anesthetic, and analgesic procedures (Sevinc et al., 2007).

### Experimental infection of goats with *B. aktasi* n. sp.

**Figure 1:**
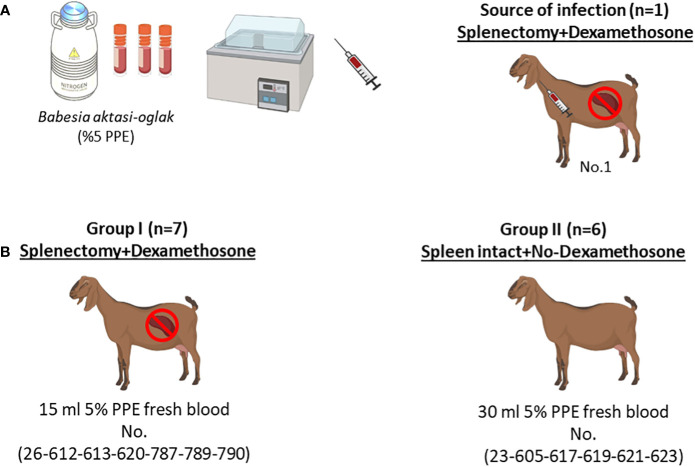
Schematic representation of the experimental infection. **(A)** Infection of the splenectomized goat (No.1) with (*B*) *aktasi* stabilate to generate the fresh infected blood for experimental infection. **(B)** Experimental infection of goats in group I and group II with fresh infected blood obtained from No.1.

Experimental infections were performed using freshly infected blood, 15 ml of *B. aktasi* n. sp. stabilate (5% PPE) (Ozubek et al., 2023). Stabilates were thawed and immediately administered intravenously to an immunosuppressed goat (No.1). When parasitemia level reached 5%, 15 ml and 30 ml of infected fresh blood taken from the donor goat were intravenously inoculated into goats in group I and group II, respectively (Guan et al., 2009) (**Figure 1**). Following the inoculation, the goats were monitored daily for signs of clinical babesiosis (fever, anemia, icterus and hemoglobinuria). Additionally, a few drops of blood from the ear tip of the goats were drawn daily, and peripheral blood smears were prepared for microscopic examination. Five milliliters of whole blood samples in anticoagulant-coated (EDTA) vacuum tubes were collected from the jugular vein of each goat, and used for DNA isolation and hematological analysis. Blood samples were also collected in non-anticoagulant tubes, and sera were separated for assessment of serum biochemical parameters.

### Microscopic detection of piroplasm

Blood smears were stained with a 10% Giemsa solution or Diff-Quick stain (DiffPlus, Biyosistem Medikal, Turkiye) and screened under 100X objective to detect intraerythrocytic piroplasm. The level of parasitemia was calculated by examining at least 20 microscopic fields as previously described (Sevinc et al., 2007).

### Haematological and biochemical parameters

Hemogram (RBC: red blood cell count; WBC: white blood cell count; HCT: hematocrit; HB: hemoglobin; PLT: platelet count; MCV: mean corpuscular volume; MCH: mean corpuscular hemoglobin; MCHC: mean corpuscular hemoglobin concentration) (Mindray Medical Electronics Co., Shenzhen, China) and serum biochemical parameters (Albumin, ALT: alanine aminotransferase, AST: aspartate aminotransferase, Creatinine, GGT: gamma glutamyl aminotransferase, Glucose, Total bilirubin, Total protein) (Fujifilm Corporation, Tokyo, Japan) were measured at four different time points tailored to each individual animal’s unique progression: first, before the start of the experiment, and subsequently, three additional times following the appearance and subsequent disappearance of piroplasm forms in each animal. Due to variations in the duration of piroplasm presence among different animals, the specific days of evaluation were adapted accordingly for each case.

### *Babesia aktasi* n. sp. PCR

Genomic DNA was isolated from 200 µl of EDTA anticoagulated blood samples from the goats using with the PureLinkTM Genomic DNA Mini Kit (Invitrogen Corporation, Carlsbad, USA) according to the manufacturer’s instructions, and stored in −20°C until use. For the detection of *B. aktasi* n. sp. DNA, a nested PCR assay was performed using two universal primers Nbab1F/Nbab1R (Oosthuizen et al., 2008) and RLBF2/RLBR2 (Georges et al., 2001).

### Data analysis

The data analysis and graphs for this study were generated using GraphPad Prism software version 8 (GraphPad Software, San Diego, CA). The software was used to display the temperature, PCV, parasitemia rate, hemogram-serum biochemistry values as the mean and standard deviation of group I, group II and negative control samples. To compare the means between the two groups, a two-tailed t test was used, with a significance level of *p*<0.05 considered to be significant. **Figure 1** was created with BioRender.com (www.biorender.com)

## Results

### *Babesia aktasi* n. sp. is highly pathogenic to immunosuppressed goats

**Figure 2:**
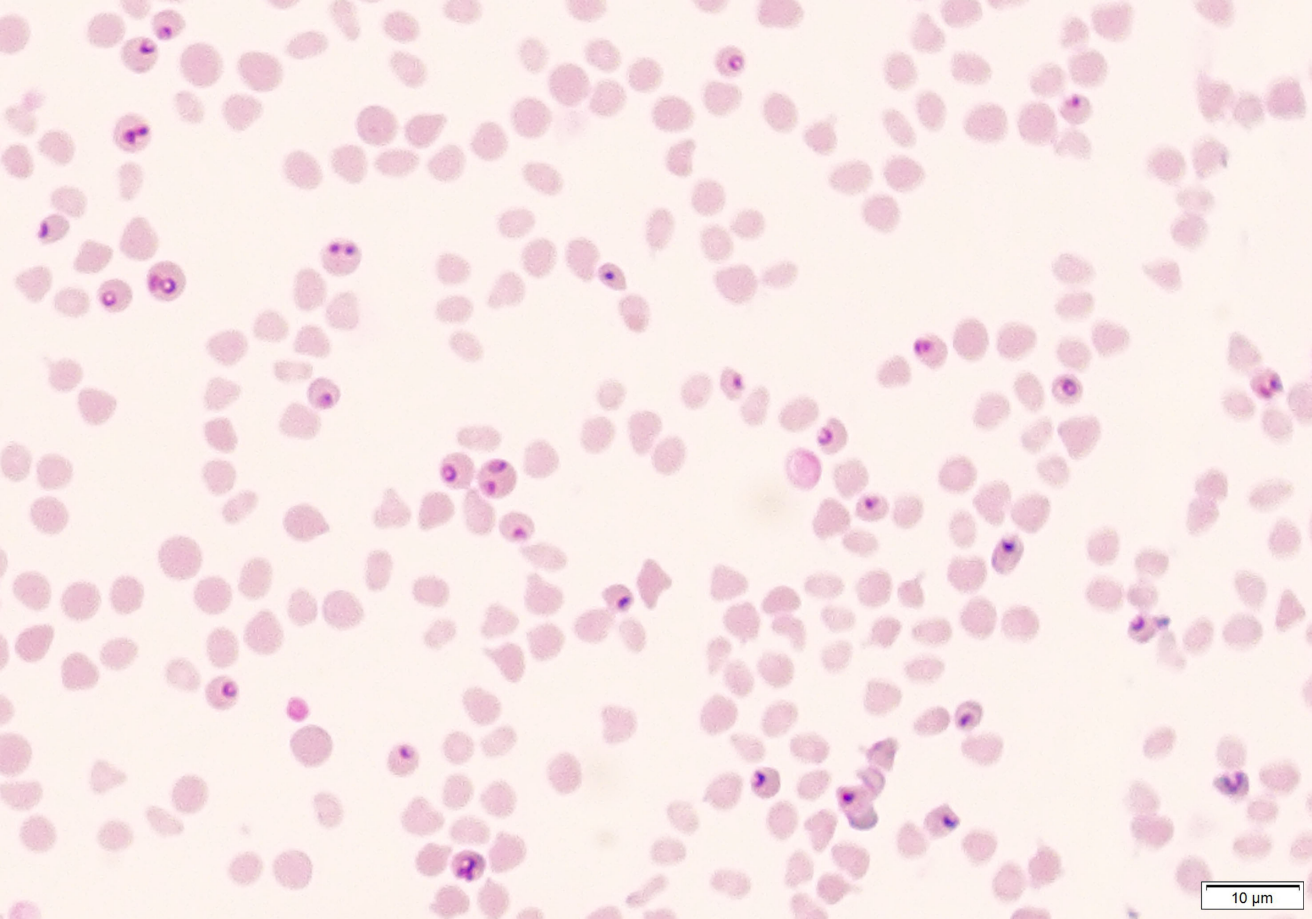
Microscopic visualization of blood smear obtained from immunosuppressed sheep (Animal ID. 787) after experimental infection with (*B*) *aktasi* n. sp.

**Figure 3:**
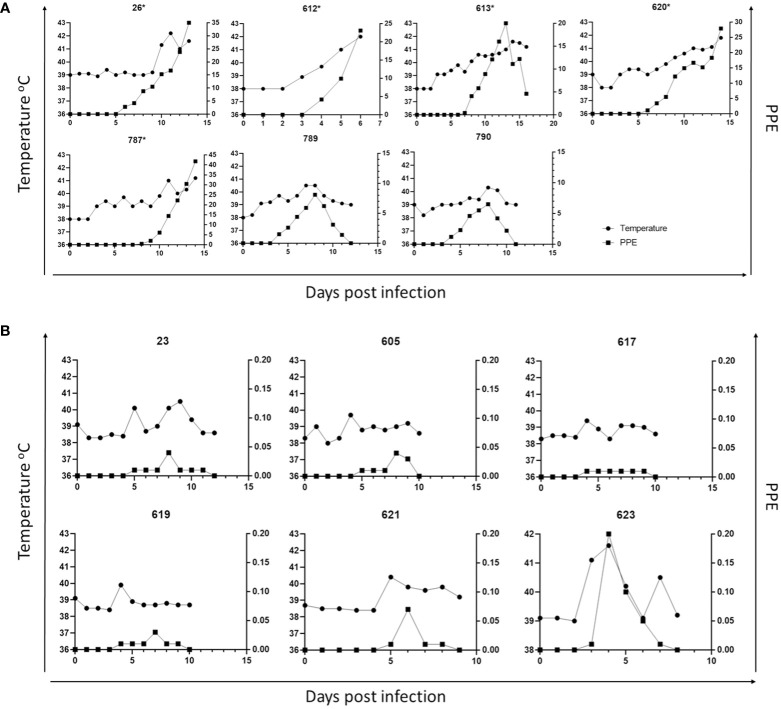
Parasitemia (PPE) and body temperature of goat in immunosuppressed **(A)** and non-immunosuppressed **(B)** groups. (* Animals humanely euthanized due to severe babesiosis caused by *B. aktasi* n. sp.).

Parasites were detected in peripheral blood between days 4 and 6 after experimental infection of immunosuppressed goats with *B. aktasi* n. sp. (**Figure 2**). As the infection progressed, all the goats developed a fever, with peak temperature ranging from 40.3°C to 42.2°C, which correlated with increased and peak parasitemia (**Figure 3**; **Table 1**). Examination of peripheral blood smears using light microscopy revealed parasitemia rates ranging from 6.5% to 41.8% (**Figure 2**; **Table 1**). Only 2 out of the 7 experimentally infected immunosuppressed goats were able to survive acute disease. Goats displaying severe clinical signs of babesiosis (26, 612, 613, 620, and 787), including symptoms such as hemoglobinuria, icterus, loss of appetite, and immobility, experienced a significant drop in hematocrit levels, falling below 10%. These goats also exhibited elevated parasitemia levels, reaching as high as 41.8%. In consideration of the severe babesiosis symptoms they were experiencing, these goats were humanely euthanized.

**Table 1:** Amount of infected blood inoculated and changes in infection parameters in group I (Splenectomy+Dexamethasone) and group II (Spleen-intact+No-Dexamethasone) goats (*humanely euthanized due to severe babesiosis caused by *B. aktasi* n. sp.).

Group I (Immunosuppressed goats)	Inoculation Type	Prepatent period (days)	Max. parasitemia (%)	Max. fever (°C)	Duration of fever days	Euthanasia (Day Post Infection)
26*	Fresh blood-15 ml	5	35	42.2	4	13
612*	Fresh blood-15 ml	3	23.1	42.2	2	7
613*	Fresh blood-15 ml	6	20	41.6	9	13
620*	Fresh blood-15 ml	5	27.9	41.8	7	14
787*	Fresh blood-15 ml	7	41.8	40.6	5	14
789	Fresh blood-15 ml	3	8.1	40.5	2	–
790	Fresh blood-15 ml	3	6.5	40.3	2	–
Group II (Non-immunosppressed)						
23	Fresh blood-30 ml	5	0.04	40.5	3	–
605	Fresh blood-30 ml	6	0.04	39.7	0	–
617	Fresh blood-30 ml	4	0.01	39.4	0	–
619	Fresh blood-30 ml	4	0.03	39.9	0	–
621	Fresh blood-30 ml	5	0.07	40.4	2	–
623	Fresh blood-30 ml	2	0.2	41.6	5	–

### Clinical and parasitological findings in experimentally infected non-immunosuppressed goats

*Babesia aktasi* n. sp. piroplasms were identified in peripheral blood of the non-immunosuppressed group of infected goats between days 2 and 5 post-inoculation, and observed until day 6 post-infection. Four out of six infected goats (23, 619, 621, 623) exhibited an increase in body temperature between days 2 and five post-infection (**Table 1**; **Figure 3**). Parasitemia ranged from 0.01% to 0.2% in peripheral blood. All the goats among the non-immunosuppressed group showed clinical signs, such as loss of appetite, stagnation, and prostration for 2-3 days, however typical symptoms of acute babesiosis, such as anemia, icterus, and hemoglobinuria, were not observed, except for an increased body temperature (**Table 1**; **Figure 3**). Notable clinical signs were observed in both immunosuppressed (group I) and non-immunosuppressed (group II) goats even though group I received only half of the parasite dose compared to animals in group II.

### *Babesia aktasi* n. sp. acute infection decreases RBC, WBC, HCT, HB, PLT, and MCHC in immunosuppressed goats

**Figure 4:**
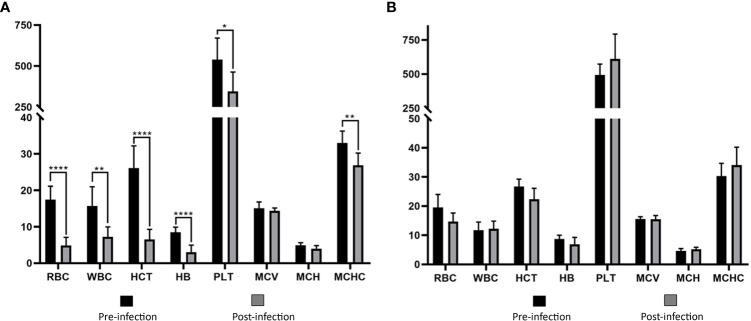
Comparison of hematological parameters in immunosuppressed **(A)** and non-immunosuppressed **(B)** goats before and after infection. Statistical significance is indicated as follows: **** *p*<0.0001, ** *p*<0.01, * *p*<0.05. RBC, red blood cell count (10^6^/μL); WBC, white blood cell count (10^3^/μL); HCT, hematocrit (%); HB, hemoglobin (g/dL); PLT, platelet count (10^3^/μL); MCV, mean corpuscular volume (fL); MCH, mean corpuscular hemoglobin (pg); MCHC, mean corpuscular hemoglobin concentration (g/dL).

**Figure 5:**
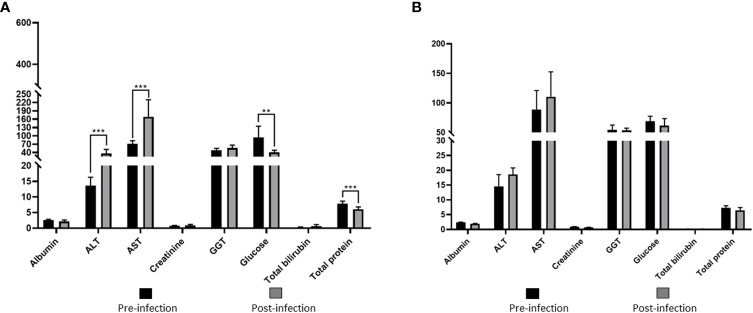
Comparison of serum biochemical parameters in immunosuppressed **(A)** and non-immunosuppressed **(B)** goats before and after infection. Statistical significance is indicated as follows: *** *p*<0.001; ** *p*<0.01. Albumine (g/dL); ALT, alanine aminotransferase (U/L); AST, aspartate aminotransferase (U/L); Creatinine (mg/dL); GGT, gamma glutamyl aminotransferase (U/L); Total bilirubin (mg/dL); Total protein (g/dL).

Values of RBC, WBC, HCT, HB, PLT, and MCHC in group I showed statistically significant decreases compared to the pre-infection levels (**Table 2**, **Figure 4**). Group II did not show any statistically significant differences in hematological and serum biochemistry parameters before and after infection. (**Table 2**). Animals in the group I had elevated levels of ALT and AST. Additionally, a decrease in glucose and total protein values was observed compared to pre-infection levels in this group (**Table 2**, **Figure 5**).

**Table 2:** Comparison of hematological and serum biochemistry parameters between group I (immunosuppressed goats) and group II (non-immunosuppressed goats).

		Group I (Immunosuppressed goats) n=7		Group II (Healthy goats) n=6	
Hematological parameters	Reference Interval*	Pre-infection	Post-infection	Pre-infection	Post-infection
RBC	8–18×10^6^/μL	17.43 ± 3.44	4.86 ± 2.06^****^	19.59 ± 4.09	14.71 ± 2.72
WBC	4–13×10^3^/μL	15.74 ± 4.87	7.26 ± 2.50^**^	11.78 ± 2.53	12.22 ± 2.43
HCT	%22–38	26.13 ± 5.63	6.53 ± 2.57^****^	26.74 ± 2.32	22.35 ± 3.49
HB	8–12 g/dL	8.48 ± 1.30	3.06 ± 1.76^****^	8.72 ± 1.21	6.87 ± 2.20
PLT	300–600× 10^3^/μL	540 ± 121.51	345.43 ± 109.5*	494 ± 73.47	612 ± 166.85
MCV	16–25 fL	15.07 ± 1.62	14.4 ± 0.7	15.54 ± 0.74	15.47 ± 1.20
MCH	5.2–8 pg	4.91 ± 0.67	4.01 ± 0.76	4.67 ± 0.71	5.18 ± 0.63
MCHC	30–36 g/dL	32.96 ± 3.09	26.86 ± 3.11^**^	30.33 ± 4.02	34.06 ± 5.71
Serum biochemistry parameters					
Albumin	2.7–3.9 g/dL	2.64 ± 0.2	2.2 ± 0.5	2.35 ± 0.1	1.94 ± 0.1
ALT	6–19 U/L	13.3 ± 2.7	45.8 ± 4.5^***^	14.5 ± 3.9	18.3 ± 2.1
AST	167–513 U/L	72.62 ± 10.8	201 ± 44.1^***^	75.62 ± 10.8	107 ± 41.2
Creatinine	1.0–1.8 mg/dL	0.8 ± 0.1	0.9 ± 0.3	0.9 ± 0.06	0.68 ± 0.08
GGT	20–56 U/L	49.3 ± 6.1	56.7 ± 10.3	53.3 ± 7.4	53.5 ± 3.7
Glucose	50–75 mg/dL	70.2 ± 8.02	41.16 ± 7.7^**^	68.2 ± 8.02	62.7 ± 11.6
Total bilirubin	0–0.1 mg/dL	0.19 ± 0.1	0.64 ± 0.5	0.12 ± 0.04	0.13 ± .002
Total protein	6.4–7.0 g/dL	7.7 ± 0.8	6.1 ± 0.7^***^	7.3 ± 0.7	6.7 ± 0.6

RBC, red blood cell count; WBC, white blood cell count; HCT, hematocrit; HB, hemoglobin; PLT, platelet count; MCV, mean corpuscular volume; MCH, mean corpuscular hemoglobin; MCHC, mean corpuscular hemoglobin concentration; ALT, alanine aminotransferase; AST, aspartate aminotransferase; GGT, gamma glutamyl aminotransferase.

(**** *p*<0.0001, *** *p*<0.001, ** *p*<0.01, * *p*<0.05).

## Discussion

Small ruminant babesiosis holds significant economic importance in the Middle East, Southern Europe, as well as in certain African and Asian countries (Yin et al., 1997; Uilenberg, 2006; Stuen, 2020; Schnittger et al., 2022; Stevanović et al., 2022), but despite its substantial impact, ovine babesiosis is still considered a neglected disease (Uilenberg, 2006; Yin et al., 2007; Stevanović et al., 2022). Furthermore, there is a lack of research on the pathogenesis of babesiosis in goats, regardless of its potential to cause fatal infections in sheep (Sevinc et al., 2013; Schnittger et al., 2022). Several parameters need to be compared to assess the pathogenicity of *Babesia* and *Theileria* species, including anemia, fever, time to peak parasitemia, duration of parasitemia, as well as hematological and serum biochemistry values (Guan et al., 2001; Bai et al., 2002; Guan et al., 2002; Shkap et al., 2007; Rahbari et al., 2008; Guan et al., 2009; Guan et al., 2010; Sevinc et al., 2013; Niu et al., 2017; Sears et al., 2022). This present study was carried out to compare the pathogenicity of *B. aktasi* n. sp. among immunosuppressed and non-immunosuppressed goats. As the behavior of *B. aktasi* in field conditions remains unknown, we have relied on previously published pathogenicity studies to design the experimental infections for our study. In determining the preferred amount of blood for the challenge dose, we have taken experimental infections conducted with *Babesia* sp. Xinjiyang, a novel pathogen discovered in China which infects small ruminants, as a reference (Guan et al., 2009). In this study, as commonly preferred in experimental *Babesia* infections in small ruminants, we opted for using young animals (under 1 year of age) that were free from tick-borne pathogens (Hasherni-Fesharki and Uilenberg, 1981; Rahbari et al., 2008; Guan et al., 2009; Guan et al., 2010). Given the distinct characteristics of various *Babesia* species, the parasitemia level in the provided blood varies across different studies, making it impractical to rely on blood parasitemia levels as a reference point. Furthermore, due to the diversity among *Babesia* species, animals that died due to infection in various studies exhibited differing levels of parasitemia. Therefore, it is challenging to establish a connection in terms of these parameters between pathogenicity studies conducted with different *Babesia* species, and meaningful correlations may not be relevant. Experimental studies in sheep and goats have confirmed the essential role of the spleen in controlling parasitemia of *Babesia* species, and consequently, removal of the spleen usually leads to uncontrolled parasite proliferation and even death (Wright and Goodger, 2018). Small ruminant *Babesia* species exhibit diverse pathogenicity and respond differently to splenectomy. For instance, experimental infection with *B. ovis* revealed a critical outcome, as all splenectomized lambs died due to severe babesiosis in two separate studies (Habela et al., 1990; Rahbari et al., 2008). In contrast, experimental infections with *B. motasi* (Uilenberg et al., 1980; Lewis et al., 1981) and *B. crassa* (Hasherni-Fesharki and Uilenberg, 1981) in sheep and goats did not result in any mortality, even after splenectomy. Regarding *Babesia* sp. Xinjiang, while splenectomized lambs survived acute infection, splenectomy combined with dexamethasone treatment resulted in death (Guan et al., 2009). In this study, severe clinical babesiosis was observed in immune suppressed goats and five out of seven animals had to be humanely euthanized on days 7 through 14 post-infection due to severe babesiosis.

Splenectomy and dexamethasone are often employed together as well-established approach in experimental studies on *B. ovata* (Fujinaga, 1982), *B. bigemina* (Ravindran et al., 2006), *Babesia* sp. Xinjiang (Guan et al., 2009) to suppress the immune system. Experimental infection of goats with *Babesia* sp. Lintan revealed that splenectomy alone did not result in any clinical signs despite the detection of high parasitemia. In the same study, when dexamethasone was administered to splenectomized goats, high parasitemia and clinical manifestations of babesiosis were observed, but no fatalities were recorded among the animals (Guan et al., 2010). These findings highlight that splenectomy alone may not be enough to trigger clinical signs in certain *Babesia* species. However, the addition of dexamethasone treatment significantly increased the likelihood of observing clinical manifestations of babesiosis (Guan et al., 2009). Therefore, in this study, the application of dexamethasone alongside splenectomy was chosen to suppress the immune system. Our main goal was to achieve a thorough suppression of the immune system, aligning with previous research that has shown enhanced results when both splenectomy and dexamethasone are used simultaneously. By employing this immunosuppression method, we could observe the genuine effects of the parasite. This approach was selected to improve the accuracy of our assessment regarding the parasite’s impact on the host. Considering the synergistic effects of splenectomy and dexamethasone in immune suppression, we found it to be the most suitable approach for our research objectives and the focal point of our study.

In this current study, four euthanized goats in the immune suppressed group exhibited severe clinical signs, such as high fever, high parasitemia, anemia, hemoglobinuria, lethargy, anorexia, rapid breathing. Our findings align with previous studies, indicating that immune suppression in lambs infected with *B. ovis* led to fever, accompanied by 70% parasitemia (Habela et al., 1990; Rahbari et al., 2008). However, in the case of experimental infections with *B. motasi*, the observed increase of temperature was mild, and the parasitemia remained at a moderate level, peaking at 6.7% (Lewis et al., 1981). Notably, splenectomized goats did not display any signs of parasitemia or clinical manifestations in an experimental infection study based on the *B. motasi* Ameland strain, (Lewis et al., 1981). Similar to *B. motasi*, experimental infections with *B. crassa* showed mild fever, ranging from 40.2°C to 41.5°C, and moderate parasitemia, reaching up to 14% (Hasherni-Fesharki and Uilenberg, 1981). These findings highlight the importance of the spleen in the immune response against *Babesia* infections and demonstrate how different *Babesia* species can lead to different clinical manifestations and parasitemia, depending on the presence or absence of the spleen (Bach et al., 2005; Buffet et al., 2011; Lewis et al., 2019; Sears et al., 2022). Interestingly, the two surviving goats also displayed similar clinical signs, but their parasitemia levels were relatively low, ranging from 6.5% to 8.1%, compared to the goats that were euthanized. Except for displaying low levels of parasitemia and a slight increase in body temperature, the group of non-immunosuppressed goats did not exhibit the typical clinical manifestations of babesiosis. The absence of babesiosis specific clinical signs, such as icterus, anemia and hemoglobinuria in spleen-intact goats suggests that the animal effectively controlled the infection and limited the progression of acute disease. Experimental infections conducted on spleen intact sheep and goats have demonstrated that various *Babesia* species can cause clinical babesiosis. For instance, in an experimental infection study with *B. ovis*, spleen-intact lambs exhibited significant parasitemia and a high mortality rate due to severe babesiosis (Habela et al., 1990; Rahbari et al., 2008). Similarly, *Babesia* sp. BQ1-Ningxian caused a low level of parasitemia in lambs but resulted in a high fever and mortality rate (Niu et al., 2017). In contrast, experimental infections with *B. motasi* and *B. crassa* in spleen-intact lambs have been reported to result in low parasitemia and mild fever (Hasherni-Fesharki and Uilenberg, 1981; Lewis et al., 1981). Additionally, experimental infection with *B. motasi* (Welsh strain) in spleen intact goats resulted in mild anemia, a modest 1% parasitemia, and mild fever (Lewis et al., 1981). Conversely, lambs infected with *Babesia* sp. Xinjiang did not exhibit any fever or symptoms (Guan et al., 2001). Interestingly, despite the high parasitemia in spleen-intact lambs experimentally infected with *Babesia* sp. BQ1-Lintan, no clinical signs were observed (Guan et al., 2010). Overall, the level of parasitemia can provide some insight into the pathogenicity of *Babesia* species, but it is not the sole determinant. Other factors, including host factors and parasite characteristics, also contribute to the clinical manifestations and severity of babesiosis (Yeruham et al., 1998; Sevinc et al., 2013). In this study, a comprehensive and objective clinical comparison was conducted to evaluate the effects of experimental infection in spleen intact goats. Our findings indicate that *B. aktasi* exhibits relatively low pathogenicity in spleen-intact goats, resulting in only mild fever and low parasitemia. This parallels the characteristics of certain other small ruminant *Babesia* species, even though some of these species can induce severe clinical infections and even mortality in spleen-intact goats.

Interpretation of clinical signs of acute babesiosis, combined with hemogram and serum biochemistry analysis, involves examination of blood tests to assess the health status of animals (Turgut, 2000). Severe clinical infection was observed in splenectomized goats in this study, resulting in a significant decrease in RBC, HCT, HB, and PLT values compared to their pre-infection levels. This decline can be attributed to the destruction of erythrocytes caused by parasite replication (Esmaeilnejad et al., 2012; Sevinc et al., 2013; Salem and Farag, 2014; Sevinc et al., 2014; Kage et al., 2019). According to hematocrit and HCT values, splenectomized goats developed severe anemia (Tvedten, 2022). In this study, MCV was normal, indicating normocytic RBC. However, MCHC showed a statistically significant decrease, indicating reduced hemoglobin concentration within RBC (hypochromic). This led to the classification of the anemia as normocytic hypochromic, where the red blood cells had normal size but lower than expected hemoglobin content. Normocytic hypochromic anemia is commonly associated with typical forms of iron deficiency (Turgut, 2000; Polizopoulou, 2010). Although we did not determine iron concentrations after experimental infection in this study, significant decreases were observed in this concentration in cattle infected with *T. annulata*, *B. bigemina* (Lotfollahzadeh et al., 2012) and *B. ovis* (Voyvoda et al., 1997) compared to uninfected animals. Various studies on anemia types in sheep babesiosis have reported macrocytic hypochromic anemia in splenectomized animals infected with *B. ovis* (Rahbari et al., 2008) and *B. motasi* (Alani and Herbert, 1988), and microcytic hypochromic anemia in spleen-intact sheep infected with *B. ovis* (Rahbari et al., 2008). However, natural infection with *B. ovis* in sheep has been associated with normocytic-normochromic anemia (Sevinc et al., 2013). In this study, thrombocytopenia, which is a significant hematological manifestation of babesiosis in dogs and sheep, was also observed. However, the decrease in HCT values, although statistically significant, was evaluated within the reference range established for goats (**Table 2**). Likewise, a significant statistical increase in the levels of ALT and AST was observed in the immunosuppressed group following experimental infection. However, the increase in AST levels remained within the reference range, similar to the HCT values (**Table 2**). Our results agree with previous reports and indicate that animals with babesiosis may experience elevated ALT levels, indicating potential disruptions in liver function associated with the disease. In particular, certain species of *Babesia* can cause liver tissue damage and potentially suppress its overall function (Rogers, 1971; Schneider et al., 2011; Sevinc et al., 2013; Esmaeilnejad et al., 2021)

In conclusion, *B. aktasi* n. sp. isolated from goats caused mild disease with minor clinical symptoms in non-immunosuppressed goats but displayed significant pathogenicity, resulting in severe clinical infections in immunosuppressed goats. A previous study revealed a 22.5% prevalence of *B. aktasi* n. sp. in clinically healthy goats, suggesting its limited pathogenicity in this host, while no evidence of *B. aktasi* n. sp. infection was found in sheep (Ulucesme et al., 2023a). Further experimental infection studies are necessary to investigate the pathogenicity of the parasite in sheep. Another study conducted in the same geographic area identified *Rhipicephalus bursa* as the predominant tick species collected from goats and suggested this tick species as the primary vector responsible for transmitting the parasite (Ulucesme et al., 2023b). This previous study was limited to local goat breeds, emphasizing the need for additional studies to encompass different breeds to expand our understanding. Future studies focused on investigating specific tick species responsible for transmitting *B. aktasi* n. sp., and in the identification of other potential hosts apart from goats that can be susceptible to infection are also needed. There is also a need for studies involving experimental infections caused by tick bites, as the tick may add additional factors that may affect the virulence of the parasite and/or the host’s susceptibility to infection. Given the dense population of mountain goats in the previous study area, it is essential to conduct further research to determine the natural reservoir of *B. aktasi* n. sp. Understanding the vector species involved in the transmission of *B. aktasi* n. sp. will provide valuable insights into the biology, epidemiology, and ecology of this pathogen.

## Data availability statement

The original contributions presented in the study are included in the article/supplementary material. Further inquiries can be directed to the corresponding author.

## Ethics statement

The animal study was approved by Firat University, Animal Experiment Ethic Committee, protocol number 2018/100. The study was conducted in accordance with the local legislation and institutional requirements.

## Author contributions

SÖ: Conceptualization, Investigation, Methodology, Project administration, Writing – original draft, Writing – review & editing. MU: Conceptualization, Investigation, Methodology, Project administration, Writing – review & editing. RB: Formal Analysis, Writing – review & editing. HA: Writing – review & editing. JL: Writing – review & editing. CS: Writing – review & editing. MA: Conceptualization, Investigation, Methodology, Project administration, Writing – review & editing.
